# Multicenter genomic analysis reveals environmental hotspots and near-clonal subclusters of *Acinetobacter baumannii* in veterinary hospitals in Beijing, China

**DOI:** 10.3389/fcimb.2026.1824342

**Published:** 2026-06-01

**Authors:** Yu Song, Bing Zhao, Siyu Chen, Rina Bai, Qi An, Qian Wang, Gege Zhang, Yang Wang, Lu Wang, Zhaofei Xia

**Affiliations:** 1 Department of Clinical Veterinary Medicine, College of Veterinary Medicine, China Agricultural University, Beijing, China; 2 Veterinary Teaching Hospital, China Agricultural University, Beijing, China; 3 National Key Laboratory of Veterinary Public Health Safety, College of Veterinary Medicine, China Agricultural University, Beijing, China; 4 Key Laboratory of Animal Antimicrobial Resistance Surveillance, Ministry of Agriculture and Rural Affairs, College of Veterinary Medicine, China Agricultural University, Beijing, China

**Keywords:** *A. baumannii*, China, environmental surveillance, risk factors, veterinary hospital

## Abstract

**Introduction:**

*Acinetobacter baumannii* is a major healthcare-associated opportunistic pathogen with exceptional environmental persistence. However, its ecological distribution and potential transmission pathways in veterinary hospital environments remain poorly characterized.

**Methods:**

Samples were collected from multiple departments and surface types across eight veterinary hospitals in Beijing from September to December 2020. A*. baumannii* isolates were recovered and subjected to antimicrobial susceptibility testing and whole-genome sequencing. Environmental risk factors were assessed using generalized linear mixed-effects models (GLMMs) with a binomial distribution and logit link, with hospital included as a random intercept and sampling site and department as fixed effects. Genomic analyses included profiling of antimicrobial resistance genes, virulence factors, and biocide/metal resistance genes, as well as Bayesian population structure and cgMLST to infer potential transmission patterns.

**Results:**

Among 1,136 environmental samples collected across eight veterinary hospitals in Beijing, 64 A*. baumannii* isolates were recovered, with an overall isolation rate of 5.63% and hospital-level rates ranging from 0.54% to 15.33%. The X-ray room showed the highest observed department-level isolation rate (16.36%, 9/55) and was significantly associated with increased *A. baumannii* recovery compared with the clinic room reference category in the multivariable GLMM. Floors (12.40%, 31/250), sinks (9.80%, 10/102), and staff uniforms (9.73%, 11/113) showed the highest surface-level isolation rates and were significantly associated with increased recovery compared with the door reference category (all *P* < 0.05). Most isolates were susceptible to the tested antimicrobial agents, while ceftriaxone showed the highest non-susceptibility rate (43.8% intermediate, 4.7% resistant). Two isolates were multidrug-resistant, including one carrying a 7,561-bp resistance region with a class 1 integron cassette array (*aadA1–cmlA–aadA2–bla*_PSE-1_). Whole-genome analysis revealed a conserved baseline resistome, core virulence determinants related to adhesion, biofilm formation, nutrient acquisition, regulation, and secretion systems, as well as broadly distributed metal tolerance and oxidative-stress defense genes. In contrast, accessory ARGs, VFs, and disinfectant- or metal-associated resistance determinants showed lineage- or isolate-specific variability. Bayesian population structure analysis resolved nine BAPS lineages with distinct spatial distribution patterns, including hospital-restricted, locally shared, and spatially dispersed lineages. cgMLST further identified hospital-specific near-clonal subclusters within these lineages, defined by ≤8 allelic differences and 0–3 core-genome SNPs. These subclusters were detected across multiple departments and environmental surfaces within the same hospital, indicating broad within-hospital environmental distribution of highly related isolates.

**Conclusions:**

This study demonstrates heterogeneous environmental contamination of *A. baumannii* in veterinary hospitals, with higher recovery from X-ray rooms, wet sites, staff uniforms, floors, and other shared or frequently contacted surfaces. Although most isolates were not multidrug-resistant, they carried conserved resistance-, virulence-, and environmental survival-associated genomic determinants, suggesting that their ecological risk may be related to surface survival, stress tolerance, and redistribution within hospital environments rather than widespread MDR clone dissemination. Population structure and cgMLST analyses revealed both inter-hospital lineage sharing and hospital-specific near-clonal subclusters, indicating the presence of highly related *A. baumannii* isolates across multiple departments and environmental surfaces within individual hospitals. These findings highlight the need to incorporate environmental opportunistic bacteria such as *A. baumannii* into veterinary hospital surveillance and infection prevention and control planning, particularly in high-use functional areas, wet zones, staff uniforms, shared equipment, and high-touch surfaces.

## 1 Introduction

*A. baumannii* is an opportunistic pathogen responsible for healthcare-associated infections, particularly in intensive care units (ICUs). Its clinical importance is amplified by its ability to persist on abiotic surfaces and to acquire antimicrobial resistance (Doughty et al., 2023; Zenati et al., 2016; Baleivanualala et al., 2024; Liu et al., 2024). In 2017, the World Health Organization (WHO) classified carbapenem-resistant *A. baumannii* (CRAB) as a critical-priority pathogen posing the greatest threat to modern medicine, highlighting its substantial global public health burden (WHO, 2017).

Studies in human hospitals indicate that *A. baumannii* persistence is closely associated with environmental reservoirs, including sinks, taps, and medical equipment (Liu et al., 2024; Doughty et al., 2023; Umezawa et al., 2015). This persistence is supported by a conserved repertoire of biofilm-associated genes, stress-response mechanisms, and metal-resistance determinants (Harding et al., 2018; Espinal et al., 2012; Boll et al., 2015; Norton et al., 2013; Aranda et al., 2011; Gayoso et al., 2014; Hassan et al., 2013; Lees-Miller et al., 2013; Juttukonda et al., 2016; Weber et al., 2017).

Pets have become integral members of many families, and close human–animal contact may facilitate the exchange of zoonotic bacteria, including antimicrobial-resistant pathogens (Walther et al., 2012; Misic et al., 2015; Pomba et al., 2017). Notably, a CRAB carrying New Delhi Metallo-β-lactamase 1 (NDM-1) was reported for the first time in a dog in Europe, raising concerns about potential human-to-animal spillover of NDM-1–producing strains (Jacobmeyer et al., 2021). Consistent with this possibility, pets and humans have been reported to share closely related *A. baumannii* clones (Nocera et al., 2021).

Veterinary hospitals are complex environments characterized by high animal turnover, frequent human-animal contact, intensive reuse of surfaces and equipment, and routine exposure to antimicrobials and disinfectants. Together, these factors may create selective pressures similar to those in human healthcare facilities, promoting the emergence and persistence of *A. baumannii* as an environmental colonizer and potential reservoir for resistance and virulence determinants. From a One Health perspective, veterinary hospitals may represent under-recognized ecological niches that contribute to the local circulation of *A. baumannii* lineages. However, the transmission dynamics and epidemiology of *A. baumannii* in veterinary hospitals remain poorly characterized (Wareth et al., 2019; Naing et al., 2022).

In this study, we conducted a cross-sectional environmental survey across eight veterinary hospitals, integrating isolation risk-factor modeling, antimicrobial susceptibility testing, whole-genome sequencing, and population-structure analysis. By combining risk assessments with genomic characterization, we aimed to elucidate the ecological drivers, resistance and virulence potential, and transmission patterns of environmental *A. baumannii* in veterinary hospital settings.

## 2 Methods

### 2.1 Sample collection

**Figure 1:**
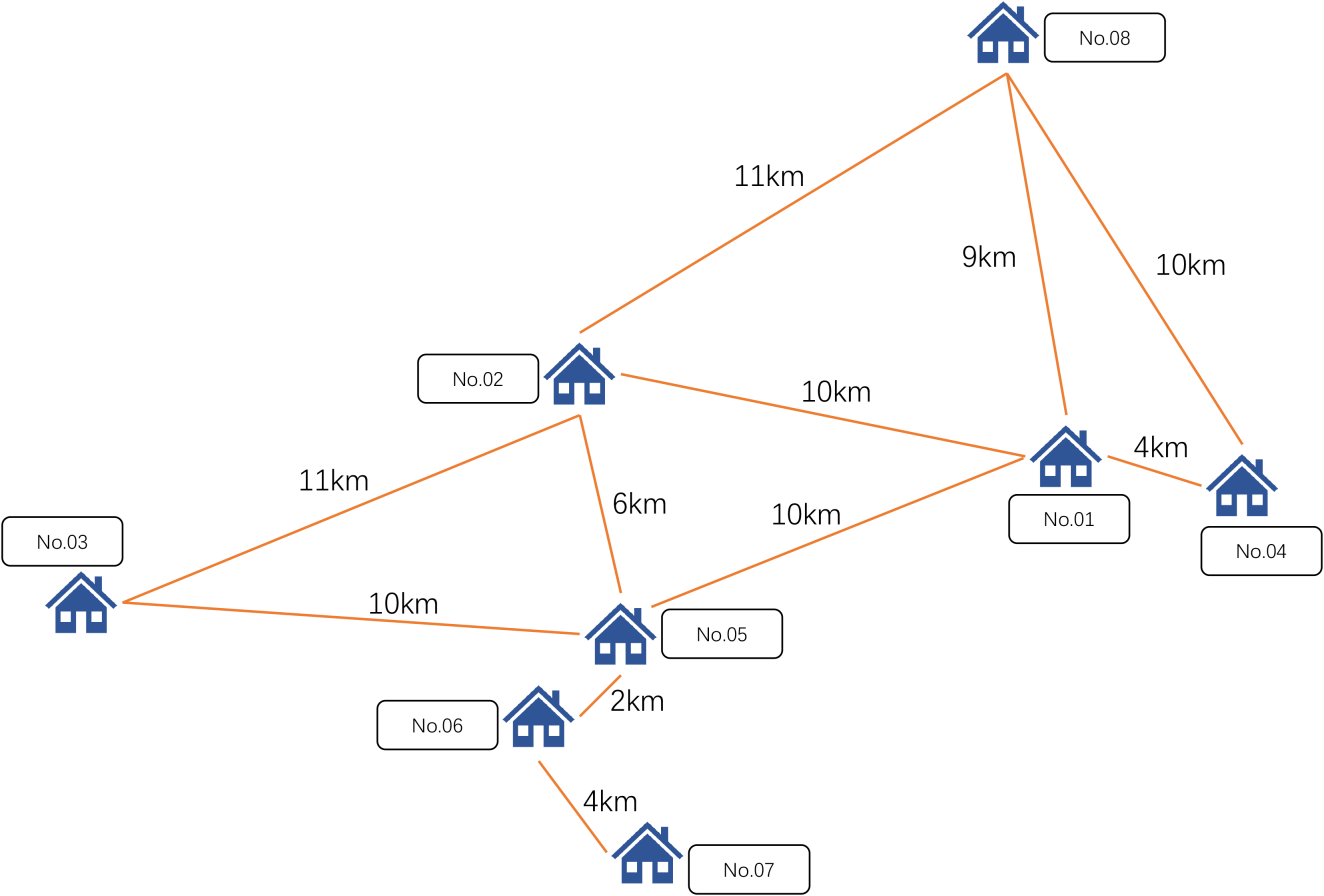
Schematic map showing the relative locations and inter-hospital distances among eight veterinary hospitals in Beijing. Black boxes indicate the hospital identification numbers (No.01–No.08), and numbers on the connecting lines indicate the geographic distances (km) between linked hospitals.

Environmental sampling was conducted in eight veterinary hospitals in Beijing, China, from September to December 2020 with institutional authorization from the participating hospitals. The spatial locations and pairwise distances among the hospitals are shown in **Figure 1**. Sampling was limited to non-living (abiotic) surfaces in clinical areas and to swabs of veterinarians’ outer clothing (cuffs, collars, and outer garment surfaces) only. No biological specimens were collected from humans or animals, and no personally identifiable information was recorded. Participating staff were informed about the purpose and procedures of sampling and provided verbal agreement prior to swabbing. Sampling locations included the pharmacy, X-ray room, ultrasound room, injection room, operating room, laboratory, ward, reception area, and consultation room. Depending on room size and the number of high-touch surfaces, 3–10 samples were collected per room. For each sample, a sterile cotton swab pre-moistened with sterile phosphate-buffered saline (PBS) was used to swab an approximately 5 × 5 cm (25 cm²) area of the target surface. Veterinarians working in the sampled rooms were sampled using the same procedure by swabbing the cuffs, collar, and outer clothing surfaces. Field negative controls were included by processing sterile PBS-moistened swabs that had not contacted any environmental surface. These negative-control swabs were transported, cultured, and processed in parallel with environmental samples, and no *A. baumannii* growth was observed.

### 2.2 Bacterial isolation and identification

All samples were cultured on CHROMID^®^ *Acinetobacter* agar plates at 37 °C for 24 h. Colonies showing the characteristic color on this medium were selected as presumptive *Acinetobacter* spp. according to the manufacturer’s instructions. To avoid repeated recovery of multiple colonies from the same original sample, only one representative colony was selected from each culture-positive sample for subsequent identification, antimicrobial susceptibility testing, and whole-genome sequencing. Species identification was initially performed by matrix-assisted laser desorption ionization–time of flight mass spectrometry (MALDI-TOF MS; Autobio, Zhengzhou, China). For confirmation, whole-genome sequencing was performed, and assembled genomes were uploaded to the JSpeciesWS server (https://jspecies.ribohost.com/jspeciesws/) for genome-based species assignment by average nucleotide identity (ANI) comparison against reference assemblies. Isolates with ANI ≥95% to *A. baumannii* representative genome assembly (GenBank assembly accession: GCA_009035845.1) were assigned as *A. baumannii* (**Supplementary Table 1**). Detailed raw sampling information, including the hospital, sampling department, sampling site, number of collected samples, and culture-positive samples, is provided in **Supplementary Table 3**.

### 2.3 Antimicrobial susceptibility testing

Broth microdilution was performed with custom-made panels (Thermo Fisher Scientific, Waltham, MA, USA) following Clinical and Laboratory Standards Institute (CLSI) document M07 (CLSI, 2018). Nine antimicrobial agents were tested for *A. baumannii*: ceftriaxone (CRO), meropenem (MEM), gentamicin (GEN), amikacin (AMK), levofloxacin (LVX), enrofloxacin (ENR), trimethoprim-sulfamethoxazole (SXT), colistin (COL), and doxycycline (DOX). *Pseudomonas aeruginosa* ATCC 27853 and *Escherichia coli* ATCC 25922 were used as quality control strains. Results were interpreted according to CLSI VET01S (CLSI, 2021) and CLSI M100 guidelines (CLSI, 2020), as applicable.

### 2.4 Statistical analysis

All statistical analyses were performed in R (v4.3.1). Statistical significance was set at *P* < 0.05.

#### 2.4.1 Isolation rate and confidence interval calculation

Isolation rates of *A. baumannii* were calculated for each hospital, department, and sampling site as the proportion of positive samples among all collected samples: *p*=*x*/*n*, where *x* is the number of positive samples and *n* is the total number of samples. Two-sided 95% confidence intervals (CIs) for proportions were calculated using the exact Clopper–Pearson method (binom.test() in R).

#### 2.4.2 Risk factor analysis using mixed-effects logistic regression

To evaluate factors associated with *A. baumannii* isolation while accounting for clustering of samples within hospitals, GLMMs with a binomial distribution and logit link were fitted using glmer() in the lme4 package. The dependent variable was sample-level isolation status (positive vs. negative).

First, to quantify between-hospital heterogeneity in isolation probability, an intercept-only random-intercept model was fitted with hospital as a random effect:


$$\text{Logit}\left\{P\left(Y_{ij}=\;1\right)\right\}=\beta_{0}+b_{0j}$$

$$b_{0j}\;∼N\;(0,\sigma^{2})$$

where *Y_ij_* denotes the isolation outcome for sample *i* from hospital *j*. Hospital-specific deviations (conditional modes) were exponentiated to obtain odds ratios relative to the overall mean.

Second, to identify sampling-related risk factors, a multivariable GLMM was fitted including sampling site and sampling department as fixed effects and hospital as a random intercept:


$$\text{Logit}\left\{P\left(Y_{ij}=1\right)\right\}=\beta_{0}+\beta_{1}Site_{ij}+\beta_{2}Dept_{ij}+b_{0j\;}$$

$$b_{0j}∼N\;\left(0,\sigma^{2}\right)$$

Reference categories were prespecified as door (sampling site) and clinic room (sampling department). Model fitting used the *bobyqa* optimizer with an increased iteration limit (maxfun = 1e5). Fixed-effect estimates were reported as odds ratios with Wald-type 95% CIs. Forest plots of odds ratios were generated using ggplot2.

### 2.5 Whole genome sequencing of *A. baumannii* and bioinformatics analysis

Genomic DNA was extracted using the HiPure Bacterial DNA Kit (Magen, Guangzhou, China). Sequencing libraries were prepared with the TruSeq Nano DNA High Throughput Library Prep Kit (Illumina, San Diego, CA, USA) and sequenced on the Illumina NovaSeq Xplus platform. Raw reads were assembled with SPAdes (v.3.14.0) (Bankevich et al., 2012) implemented in the Unicycler (v.0.5.0) (Wick et al., 2017). To assess whether highly similar genomes represented technical or sampling duplicates, genome dereplication was performed using dRep (Olm et al., 2017). Genomes were clustered using stringent thresholds of 99.99% ANI and 99% aligned fraction. dRep clustering results were further interpreted together with sampling metadata, including hospital, sampling department, sampling site, and environmental surface. Because only one representative colony was selected from each culture-positive sample, genomes from different sampling sites were not automatically removed solely based on dRep clustering, but were interpreted as repeated recovery of near-identical environmental clones when supported by distinct sampling origins. The dRep results are provided in **Supplementary Table 1**.

Genomes were annotated with Prokka (Seemann, 2014). Core-genome single nucleotide polymorphism (SNP) alignment was generated using Parsnp (v.2.0.2) (Todd et al., 2014). Recombinant regions were filtered during Parsnp analysis using the -x option, and the core-genome SNP phylogenetic tree was inferred from the recombination-filtered alignment. Pairwise core-genome SNP distances were calculated from the recombination-filtered SNP alignment using snp-dists (**Supplementary Table 2**). The phylogeny was midpoint-rooted and visualized with iTOL (https://itol.embl.de/). Population structure was assessed using RhierBAPS (Tonkin-Hill et al., 2018; Cheng et al., 2013). Multilocus sequence typing (MLST) was determined via PubMLST (https://pubmlst.org/mlst). Core-genome MLST (cgMLST) allele calling was performed using chewBBACA (AlleleCall module) with a curated *A. baumannii* cgMLST schema (Silva et al., 2018). Allele profiles were used to construct minimum spanning trees (MSTs) in PHYLOViZ Online (https://online.phyloviz.net/). Antimicrobial resistance genes (ARGs), virulence factors (VFs), and biocide/metal resistance genes (MRGs) were identified using ABRicate (v1.0.1) against the NCBI AMR database (Feldgarden et al., 2019), the Virulence Factor Database (VFDB) (Chen et al., 2016), and BacMet (Pal et al., 2014). Gene neighborhood maps were generated from Prokka annotations.

## 3 Results

### 3.1 Risk factors associated with the environmental prevalence of *A. baumannii* in veterinary hospitals

**Figure 2:**
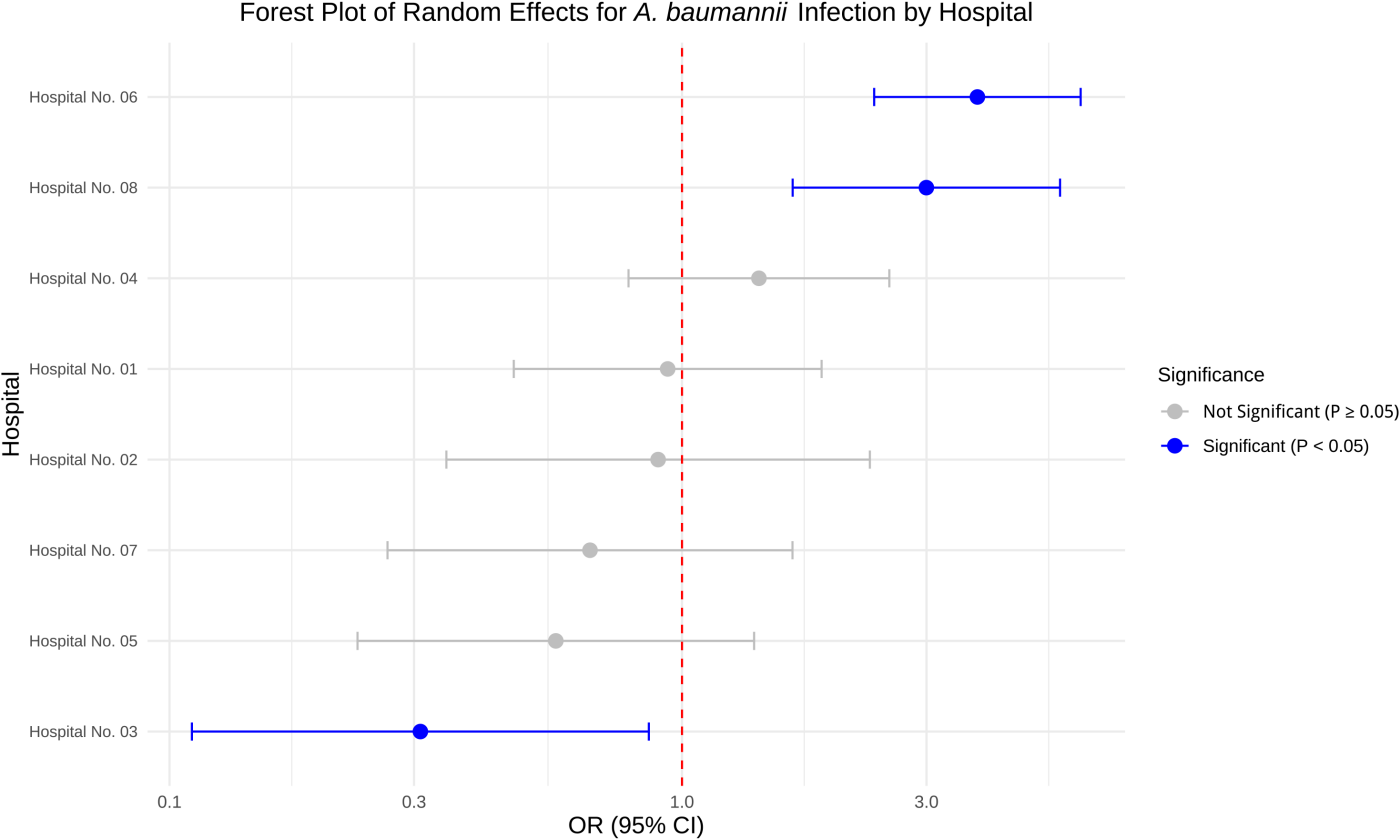
Differences in environmental *A. baumannii* Isolation Rates among Eight Veterinary Hospitals in Beijing (Sep–Dec 2020).

A total of 1,136 environmental samples were collected from eight veterinary hospitals, yielding 64 A*. baumannii* isolates. The overall isolation rates ranged from 0.54% to 15.33% across veterinary hospitals. The highest recovery rates were observed in Hospital No. 6 (15.33%, 21/137) and Hospital No. 8 (13.04%, 12/92), whereas Hospital No. 3 exhibited the lowest rate (0.54%, 1/184) (**Table 1**). Using the intercept-only random-intercept GLMM described in Section 2.4.2, with hospital included as a random effect, hospitals No. 6 and No. 8 had significantly higher isolation rates than the overall mean, while Hospital No. 3 had a significantly lower rate (*P* < 0.05; **Figure 2**).

**Table 1:** Environmental isolation rates of *A. baumannii* in eight veterinary hospitals in Beijing (Sep–Dec 2020).

Hospital ID	Number of samples	Number of *A. baumannii* Isolates	Isolation rate (with 95% CI)
1	180	7	3.89% (1.58–7.85%)
2	83	3	3.61% (0.75–10.20%)
3	184	1	0.54% (0.01–2.99%)
4	182	12	6.59% (3.45–11.23%)
5	153	4	2.61% (0.72–6.56%)
6	137	21	15.33% (9.75–22.47%)
7	125	4	3.20% (0.88–7.99%)
8	92	12	13.04% (6.93–21.68%)
Total	1136	64	5.63% (4.37–7.14%)

**Figure 3:**
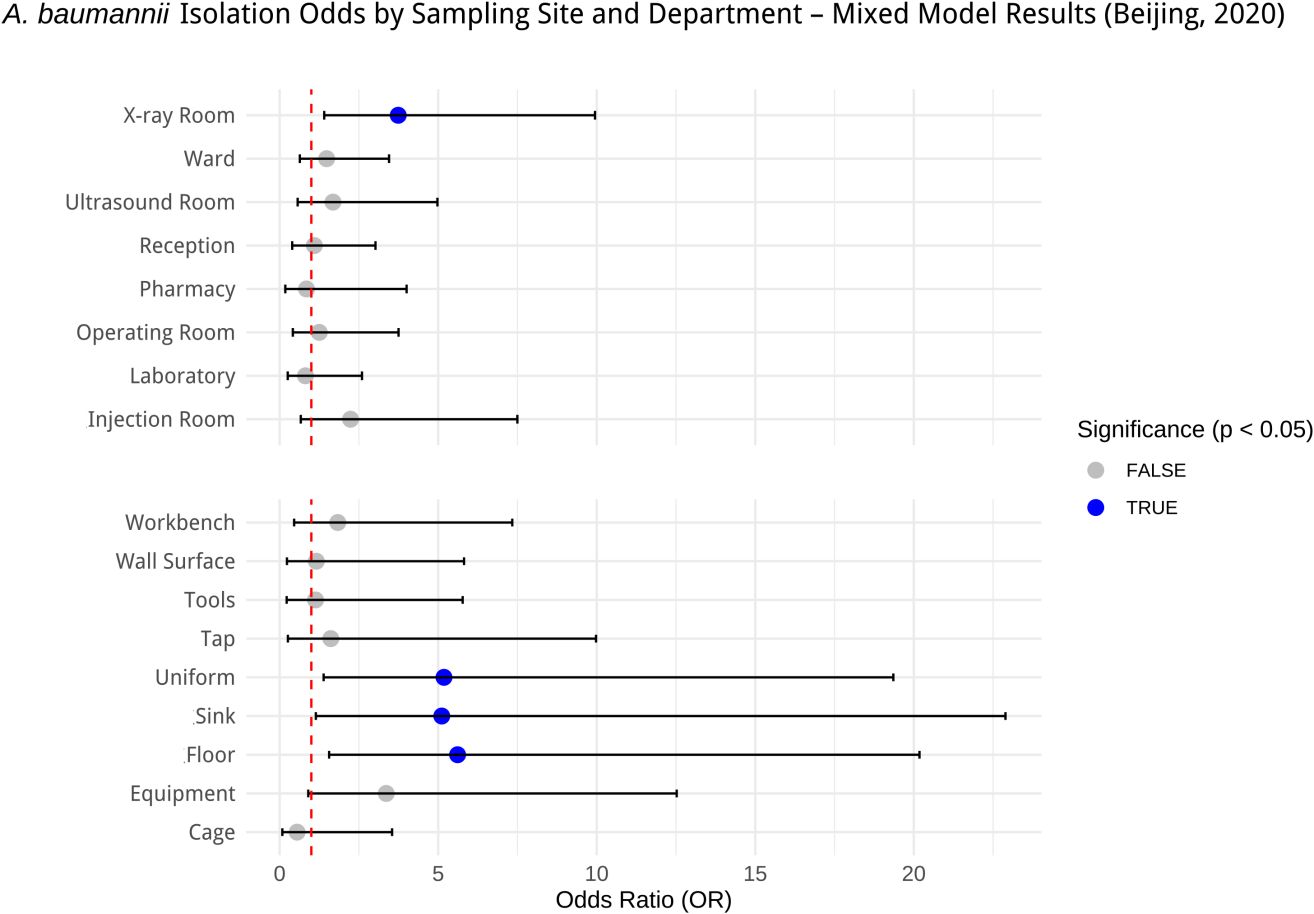
Differences in environmental isolation rates of *A. baumannii* across sampling sites and departments in 8 veterinary hospitals in Beijing (Sep–Dec 2020).

Among nine sampling locations, the largest absolute numbers of isolates were recovered from the clinic room (15/295) and ward (14/312), reflecting the larger number of samples collected in these departments. However, when standardized by sample number, the X-ray room showed the highest isolation rate (16.36%, 9/55), followed by the ultrasound room (7.14%, 5/70) and the injection room (6.35%, 4/63). All other departments had rates below 6% (**Table 2**). Using the multivariable GLMM described in Section 2.4.2, with clinic room prespecified as the reference category for sampling department, the X-ray room was associated with a significantly higher *A. baumannii* recovery rate than the clinic room reference category (*P* < 0.05; **Figure 3**).

**Table 2:** Environmental isolation rates of *A. baumannii* across different sampling departments in eight veterinary hospitals in Beijing (Sep–Dec 2020).

Sampling department	Number of samples	Number of *A. baumannii* isolates	Isolation rate (with 95% CI)
Pharmacy	51	2	3.92% (0.48–13.46%)
X-ray Room	55	9	16.36% (7.77–28.80%)
Injection Room	63	4	6.35% (1.76–15.47%)
Ultrasound Room	70	5	7.14% (2.36–15.89%)
Operating Room	86	5	5.81% (1.91–13.05%)
Laboratory	93	4	4.30% (1.18–10.65%)
Reception	111	6	5.41% (2.01–11.39%)
Clinic Room	295	15	5.08% (2.87–8.25%)
Ward	312	14	4.49% (2.47–7.41%)
Total	1136	64	5.63% (4.37–7.14%)

Marked heterogeneity was also observed across ten surface types. The highest isolation rates were detected on floors (12.40%, 31/250), sinks (9.80%, 10/102), and staff uniforms (9.73%, 11/113) (**Table 3**). In the same multivariable GLMM, floors, sinks, and staff uniforms were significantly associated with increased *A. baumannii* isolation rate compared with the door reference category (*P* < 0.05; **Figure 3**).

**Table 3:** Environmental isolation rates of *A. baumannii* across different sampling sites in eight veterinary hospitals in Beijing (Sep–Dec 2020).

Sampling site	Number of samples	Number of *A. baumannii* isolates	Isolation rate (with 95% CI)
Tap	59	2	3.39% (0.41–11.71%)
Cage	137	2	1.46% (0.18–5.17%)
Wall Surface	107	3	2.80% (0.58–7.98%)
Door	136	3	2.21% (0.46–6.31%)
Sink	51	5	9.80% (3.26–21.41%)
Tools	108	5	4.63% (1.52–10.47%)
Workbench	152	7	4.61% (1.87–9.26%)
Staff Uniform	113	11	9.73% (4.96–16.75%)
Equipment	152	11	7.24% (3.67–12.58%)
Floor	121	15	12.40% (7.11–19.62%)
Total	1136	64	5.63% (4.37–7.14%)

### 3.2 Antimicrobial susceptibility profiles of *A. baumannii* in veterinary hospital environmental isolates

**Figure 4:**
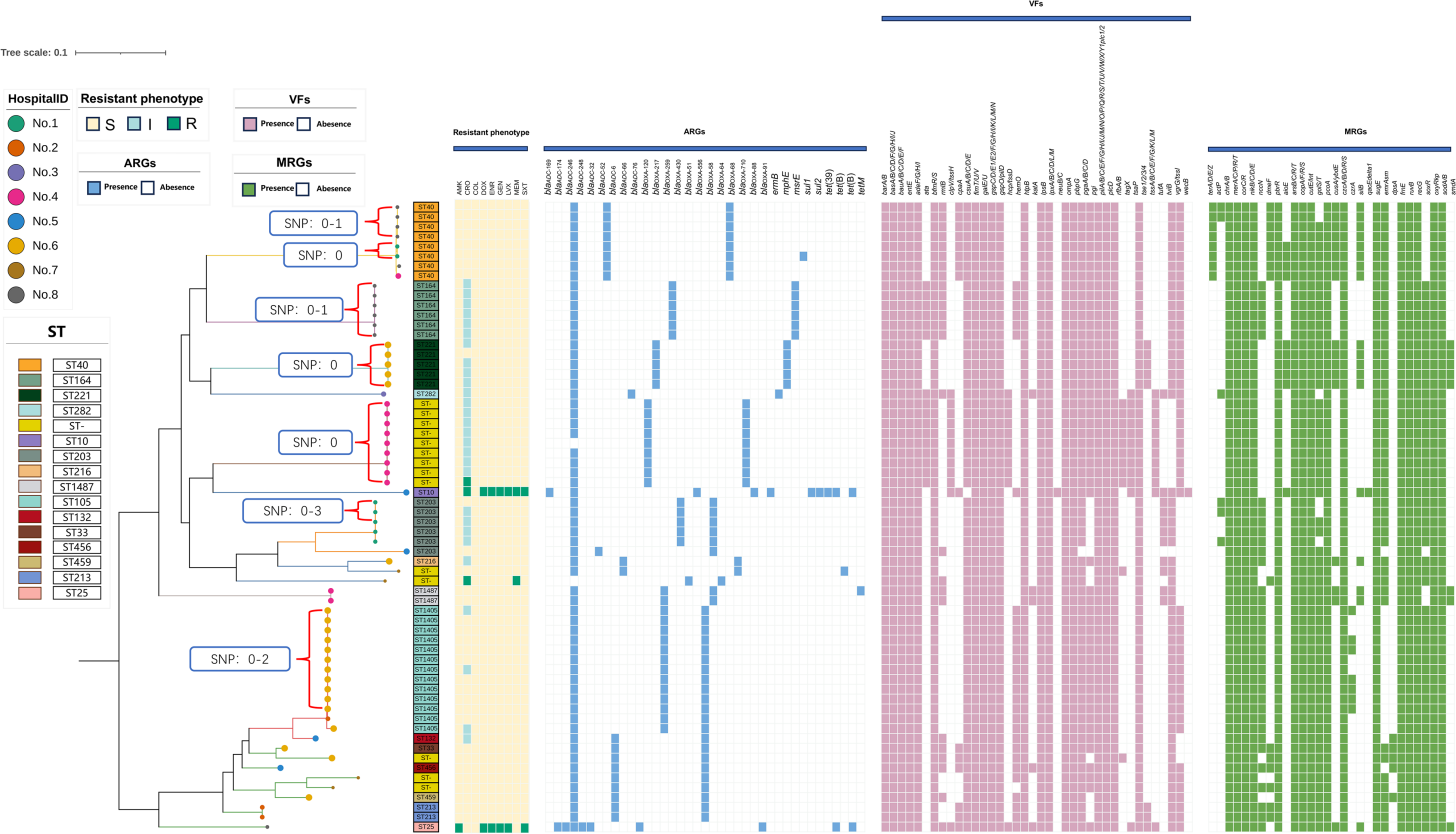
Core-genome phylogenetic tree of the *A. baumannii* in veterinary hospital environmental isolates. ARGs: antimicrobial resistance genes. VFs: virulence factors. MRGs: biocide/metal resistance genes. Isolates marked with red brackets represent closely related strains with SNP differences ranging from 0 to 3; the corresponding SNP differences are indicated in the adjacent blue boxes. ST- denotes novel sequence types that have not yet been registered in the MLST database.

Overall, most isolates remained highly susceptible to the tested antimicrobial agents, although a subset exhibited pronounced resistance phenotypes. CRO showed the highest rate of non-susceptibility, with 43.8% (28/64) of isolates classified as intermediate and 4.7% (3/64) as resistant. In contrast, all isolates were susceptible to COL. Notably, two isolates displayed resistance to multiple antimicrobial classes, including β-lactams, fluoroquinolones, aminoglycosides, and tetracyclines, and were classified as MDR (**Figure 4**).

### 3.3 Integrated resistance and virulence profiles of the *A. baumannii* in veterinary hospital environmental isolates

**Figure 5:**
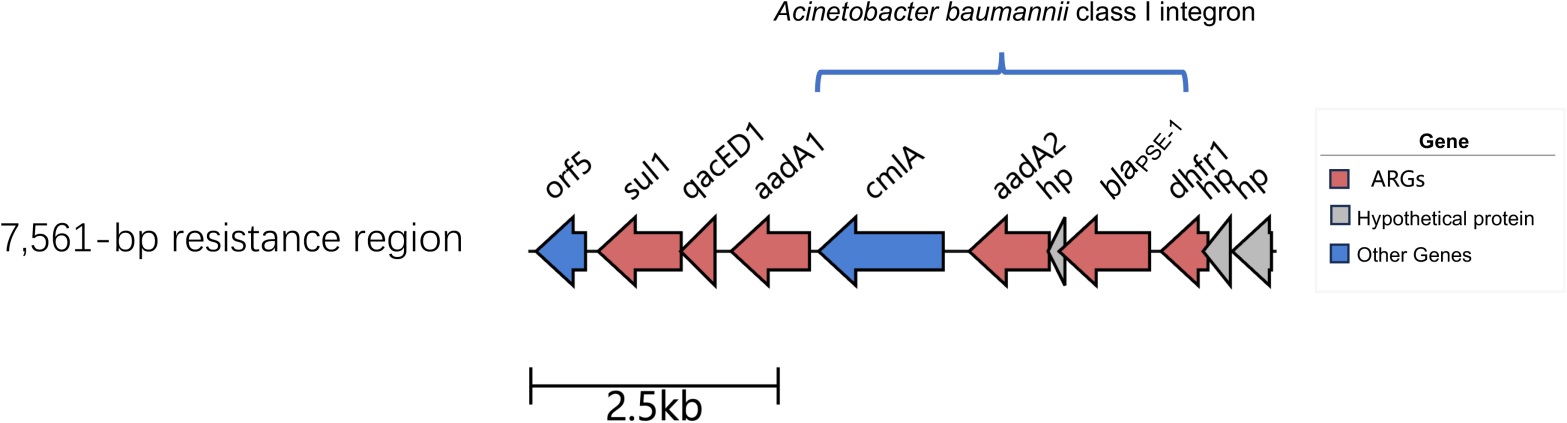
Genetic environments of class 1 integron genes among *A. baumannii* in veterinary hospital environmental isolates.

Whole-genome analysis of the 64 A*. baumannii* isolates revealed a largely conserved resistome with lineage-specific variability. In total, 40 ARGs conferring resistance to nine antibiotic classes were detected, with aminoglycoside and β-lactam resistance determinants being predominant. The aminoglycoside resistance gene *ant(3'')-IIa* was almost ubiquitous (96.9%). Endogenous β-lactamase variants were widely detected but exhibited heterogeneous distribution: multiple *bla*_ADC_ and *bla*_OXA_ alleles were identified, with individual variants (*bla*_ADC-155_, *bla*_ADC-156_, *bla*_ADC-246_, *bla*_ADC-32_, *bla*_OXA-120_, *bla*_OXA-217_, *bla*_OXA-430_, *bla*_OXA-556_) each detected at frequencies of 10.0%–35.0% (**Figure 4**). In contrast, ARGs conferring resistance to sulfonamides, macrolides, and tetracyclines were largely restricted to the two MDR isolates (**Figure 4**). Notably, one MDR isolate carried a 7,561-bp resistance region harboring a class 1 integron cassette array (*aadA1*–*cmlA*–*aadA2*–*bla*_PSE-1_) (**Figure 5**).

Virulence profiling indicated that most isolates carried a conserved core virulence gene set, with detection rates exceeding 78.0%. These genes are primarily involved in adherence, biofilm formation, immune modulation, nutrient acquisition, regulation, and secretion systems, and include *fimT/U/V, csuA–E, pgaA–D, gspC/D/E1/E2/F/G/H/I/K/L/M/N, vgrG/tssI, ompA, lpxA/B/C/D/L/M, galE/U, barA/B*, and *bfmR/S* (**Figure 4**). In contrast, several accessory virulence genes—particularly those related to type VI secretion system components, exotoxins, exoenzymes, stress survival, and surface adhesion—exhibited marked isolate-specific variability (1.5%–40.0%).

In parallel, screening of MRGs suggested broad capacity for heavy-metal tolerance and oxidative stress defense. Genes associated with resistance to multiple metals (e.g., chromium, mercury, arsenic, copper, nickel, and cobalt), together with oxidative stress regulators (*soxR, oxyRkp, sodA/B*), were detected in all isolates. Additional determinants, including *qacEdelta1*, *pcoA*, *czcA/B/D/R/S*, and *silB*, showed variable distributions (**Figure 4**).

### 3.4 Phylogenetic analysis of *A. baumannii* in veterinary hospital environmental isolates

**Figure 6:**
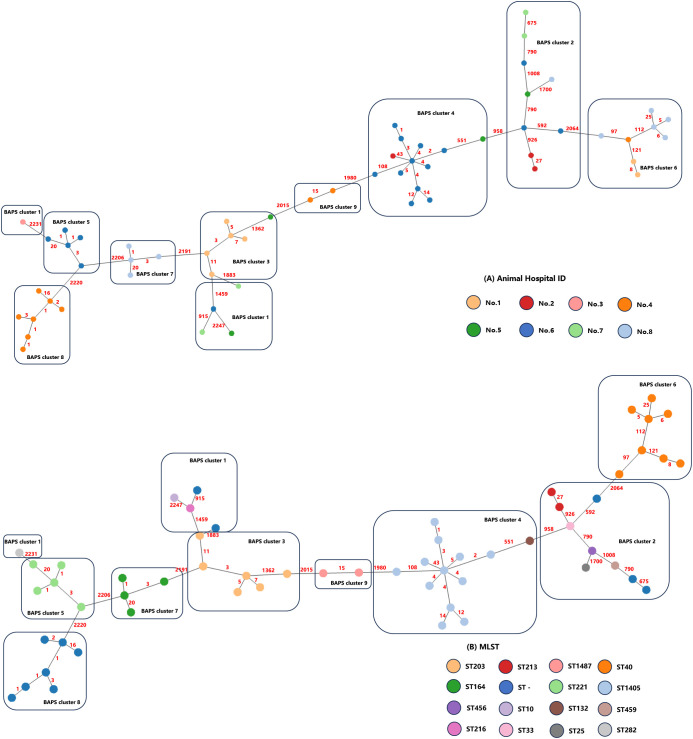
Minimum spanning tree (MST) based on the cgMLST allelic profiles of 64 A*. baumannii* isolates from veterinary hospital environments in Beijing. Each circle represents an allelic profile derived from 2,390 core gene of the cgMLST. Red numbers next to the connecting lines indicate the number of genes with different alleles. Black boxes highlight the BAPS clusters identified by Bayesian population structure analysis. **(A)** Circle colors represent different animal hospitals. **(B)** Circle colors represent MLST sequence types (STs) based on the Pasteur scheme. ST- denotes novel sequence types that have not yet been registered in the MLST database.

By integrating the BAPS clustering results (**Figure 6**) with the spatial distribution of veterinary hospitals (**Figure 1**), three major spatial distribution patterns of *A. baumannii* lineages were identified among the hospitals. The first pattern comprised hospital-restricted lineages, which were detected in only one hospital, indicating a relatively localized distribution. Specifically, BAPS cluster 5 was found only in hospital No. 6, BAPS cluster 7 was detected only in hospital No. 8, and BAPS clusters 8 and 9 were both restricted to hospital No. 4.

The second pattern comprised locally shared lineages within nearby hospitals or within the same spatial branch. These lineages were mainly distributed among hospitals that were geographically close or located within the same branch of the hospital network. For example, BAPS cluster 4 was detected in both hospital No. 5 and hospital No. 6, which were only 2 km apart. BAPS cluster 6 was detected in hospital No. 1, hospital No. 4, and hospital No. 8, all of which were located within the right-side spatial branch. Among these hospitals, hospital No. 1 and hospital No. 4 were relatively close, with a distance of 4 km, whereas hospital No. 8 was 9 km and 10 km away from hospital No. 1 and hospital No. 4, respectively. This distribution suggests that BAPS cluster 6 was mainly associated with the right-side hospital branch. BAPS cluster 1 was detected in hospital No. 3, hospital No. 5, hospital No. 6, and hospital No. 7. Among these, hospital No. 5, hospital No. 6, and hospital No. 7 formed a relatively compact lower-left branch, with pairwise distances of 2–4 km. In addition, this cluster extended to hospital No. 3, which was 10 km from hospital No. 5, indicating that its distribution was centered on the lower-left neighboring branch with some degree of spatial extension.

The third pattern comprised cross-branch or spatially dispersed lineages. These lineages were not limited to a single hospital or to neighboring hospitals within the same branch, but were instead detected across more spatially separated hospital nodes. BAPS cluster 3 was detected in hospital No. 5 and hospital No. 1, which were 10 km apart and located in the lower-left branch and the middle-right region of the network, respectively. BAPS cluster 2 showed a broader spatial distribution and was detected in hospital No. 2, hospital No. 5, hospital No. 6, hospital No. 7, and hospital No. 8, covering the central connecting node, the lower-left neighboring branch, and the upper hospital node. This pattern indicates that BAPS cluster 2 had a more widely distributed presence across the hospital network.

Integration of cgMLST analysis revealed multiple tightly connected subclusters within the BAPS lineages, defined by within-cluster distances of ≤8 allelic differences (**Figure 6**) and 0–3 core-genome SNPs (**Figure 4**). These subclusters included eleven ST1405 isolates and five ST221 isolates from hospital No. 6, four ST40 isolates and six ST164 isolates from hospital No. 8, three ST203 isolates and two ST40 isolates from hospital No. 1, and nine ST- isolates from hospital No. 4. Each of these groups formed a hospital-specific subcluster within its corresponding BAPS lineage. Notably, several BAPS clusters containing these hospital-specific subclusters also included isolates from other hospitals (**Figure 6**). Consistently, dRep analysis assigned these closely related isolates to the same highly similar genome clusters (**Supplementary Table 1**), although dRep clustering alone was not used to define technical duplicates. Because only one representative colony was selected from each culture-positive sample, these highly related genomes did not represent repeated sequencing of multiple colonies recovered from the same original swab sample. Further integration of sampling department and sampling site information showed that most of these hospital-specific subclusters were not restricted to a single sampling point, but were distributed across multiple functional areas, including clinic rooms, wards, operating rooms, X-ray rooms, ultrasound rooms, laboratories, pharmacies, injection rooms, and reception areas, as well as diverse environmental surfaces, such as staff uniforms, equipment, floors, workbenches, tools, sinks, taps, doors, and cages, within the same hospital (**Supplementary Table 1**). These findings suggest repeated environmental recovery of near-identical clones across different hospital microenvironments rather than technical duplication from the same original sample. Pairwise comparisons of closely related isolates within each subcluster showed that MRG profiles were the most conserved among the analyzed gene-profile categories (**Figure 4**). Among the seven subclusters, four had completely identical MRG profiles, whereas the remaining subclusters differed by only one to two MRG categories, corresponding to 3.33%–6.67% of the 30 MRG categories analyzed. In contrast, ARG and VF profiles showed a certain degree of subcluster-dependent variation, with overall mean pairwise differences of 7.49% and 6.73%, respectively, and maximum differences of 28.21% and 36.84%, respectively. Antimicrobial resistance phenotypes were generally similar within most subclusters, with phenotypic variation mainly involving CRO. However, individual isolates with broader resistance profiles were observed in the hospital No. 1-ST203 and hospital No. 8-ST164 subclusters, resulting in relatively higher phenotypic heterogeneity in these two groups. Overall, these isolates showed high concordance in cgMLST clustering, core-genome SNP differences, ST/BAPS assignment, hospital origin, and MRG profiles, while exhibiting limited or subcluster-specific variation in ARGs, VFs, and antimicrobial resistance phenotypes.

## 4 Discussion

We found that environmental hotspots of *A. baumannii* in veterinary hospitals differ from those commonly reported in human healthcare settings. Although clinic rooms and wards yielded larger absolute numbers of isolates because of greater sampling intensity, the X-ray room showed the highest observed isolation rate and was significantly associated with increased recovery compared with the clinic room reference category in the multivariable GLMM. In human hospitals, *A. baumannii* contamination often concentrates around bed units and respiratory-support equipment, consistent with a “bedside local circulation” pattern (Liu et al., 2024). In contrast, in veterinary hospitals, higher recovery rates were observed in high-turnover functional areas, X-ray rooms, and on wet sites, textile carriers, and shared surfaces or items, including sinks, floors, and staff uniforms. Together, these findings suggest that frequently used and highly connected locations and fomites may contribute to the redistribution of *A. baumannii* across functional areas. Existing infection prevention and control guidance for small animal veterinary clinics provides an important framework for practice-level biosecurity (Anderson et al., 2008). However, the disease- and agent-specific precaution table in this guidance does not specifically address *A. baumannii*. Previous studies have shown that *A. baumannii* is an opportunistic pathogen responsible for healthcare-associated infections, particularly in ICUs (Doughty et al., 2023). Therefore, by identifying *A. baumannii*-associated risk locations in veterinary hospital environments, the present study may help further refine current IPC priorities.

In this study, BAPS clustering results were integrated with the spatial distribution of veterinary hospitals, revealing clear differences in the spatial distribution of different *A. baumannii* genetic lineages among hospitals. Some lineages were restricted to a single veterinary hospital, whereas others were shared among geographically close hospital branches or were distributed across more spatially dispersed hospital nodes. Similar lineage-specific distribution patterns have also been reported in studies of carbapenem-resistant *A. baumannii* (CRAB) in human ICUs. In one such study, CRAB genetic lineages were defined using a Bayesian approach, and some lineages were associated with only a single patient, suggesting sporadic occurrence. In contrast, other lineages were repeatedly detected in multiple patients and their bed-unit environments, with relatively small within-lineage cgSNP differences; based on the available temporal, spatial, patient-bed, and environmental sampling information, that study inferred recent ICU-associated dissemination and environmental involvement. The same lineages were identified in adjacent bed units, adjacent rooms, and consecutive bed-unit occupants, and many putative dissemination events were consistent with environmental contamination. In some cases, patients who were CRAB-negative at admission later yielded lineages that had already been present in their bed-unit or room environments, indicating that contaminated environments may serve as an important source of CRAB acquisition (Doughty et al., 2023).

The local sharing or spatially dispersed distribution of different BAPS lineages among veterinary hospitals observed in the present study is partially consistent with the heterogeneous lineage distribution patterns reported in the ICU study. These findings suggest the possibility of local persistence, regional dissemination potential, inter-hospital lineage sharing, or common-source introduction in veterinary hospital settings. However, without longitudinal sampling and epidemiological linkage data, these spatial patterns should be interpreted as evidence of shared genetic lineages among hospitals rather than definitive inter-hospital transmission. Future studies should combine time-series sampling, paired animal clinical and hospital environmental isolates, case movement information, and higher-resolution SNP analysis to clarify whether these lineage-sharing patterns reflect persistent environmental contamination, repeated introduction, common-source exposure, or actual hospital-associated dissemination.

For *A. baumannii*, isolates differing by 0–8 cgMLST alleles or by no more than 3 SNPs have been proposed to represent highly related strains, potentially associated with recent outbreak-related clusters (Higgins et al., 2017; Schurch et al., 2018). In the present study, the hospital-specific subclusters were supported by both cgMLST and core-genome SNP analyses, with isolates differing by ≤8 cgMLST alleles and only 0–3 core-genome SNPs. Such low genetic distances indicate extremely close genetic relatedness and suggest that these isolates may represent the same clone or recently diverged clonal populations. Because only one representative colony was selected from each culture-positive sample, these near-identical genomes were unlikely to represent repeated sequencing of multiple colonies from the same original swab sample. Notably, these near-clonal isolates were not confined to a single sampling point or a specific functional area, but were detected across multiple departments and diverse environmental surfaces within the same hospital. Therefore, we interpret these patterns as repeated environmental recovery of near-identical clones across different hospital microenvironments, rather than simple technical duplication from the same original sample. Similar observations have been reported in human healthcare settings. Previous studies have recovered CRAB from high-touch ICU surfaces, such as infusion pump screens and emergency trolleys, and showed that these environmental isolates were genetically identical or highly related to patient-derived outbreak clones, with SNP differences ranging from 0 to only a few SNPs (Baleivanualala et al., 2024). These findings indicate that hospital environmental surfaces can harbor near-identical *A. baumannii* clones related to clinical outbreak populations and may contribute to their environmental persistence or redistribution within healthcare environments. Although the present study did not include paired animal clinical isolates and lacked longitudinal sampling and detailed epidemiological linkage data, the detection of highly related subclusters across multiple departments and environmental surfaces suggests that veterinary hospital environments may provide ecological niches for the short-term persistence and local expansion of *A. baumannii*.

The genomic characteristics of these subclusters further support this interpretation. Among the analyzed gene-profile categories, MRG profiles were the most conserved. Four of the seven hospital-specific subclusters had completely identical MRG profiles, whereas the remaining subclusters differed by only one to two MRG categories. This high level of conservation suggests that metal- and biocide-associated resistance determinants may constitute a relatively stable background in these closely related clonal populations. These genes may contribute to bacterial survival under environmental stress, including exposure to disinfectants, metals, cleaning agents, or other surface-associated selective pressures. In veterinary hospitals, environmental cleaning, disinfectant use, water-associated sites, animal cages, shared equipment, and high-touch surfaces coexist; therefore, conserved MRG profiles may provide an ecological advantage that enables these strains to persist across different hospital microenvironments.

Previous studies have shown that the ecological success of *A. baumannii* in healthcare environments is closely linked to traits associated with persistence, colonization, and environmental survival (Harding et al., 2018). These traits are supported by multiple determinants, including capsule and outer-membrane components, desiccation-associated DNA damage repair, oxidative-stress tolerance, and adaptation to disinfectant pressure (Espinal et al., 2012; Boll et al., 2015; Norton et al., 2013; Aranda et al., 2011; Gayoso et al., 2014; Hassan et al., 2013). In addition, functions related to host interaction and microbial competition, such as surface glycoconjugates, micronutrient acquisition, and secretion systems, have also been implicated in environmental survival and niche adaptation (Lees-Miller et al., 2013; Russo et al., 2010; Juttukonda et al., 2016; Weber et al., 2017). Consistent with these findings, isolates in the present study commonly carried conserved determinants related to adhesion, biofilm formation, secretion systems, regulation, metal tolerance, and oxidative-stress defense. Therefore, the recovery of highly related isolates from multiple environmental surfaces and hospital departments may reflect the ability of *A. baumannii* to survive on dry surfaces, tolerate environmental stress, form biofilms, and survive on shared equipment or high-touch surfaces.

At the same time, variation in ARG, VF, and antimicrobial resistance phenotypes indicates that these closely related subclusters were not completely identical in accessory gene content or phenotype. Compared with MRG profiles, ARG and VF profiles showed more evident subcluster-dependent variation, and antimicrobial resistance phenotypes were generally similar but not fully uniform. This was particularly evident in the hospital No. 1-ST203 and hospital No. 8-ST164 subclusters, where individual isolates showed broader resistance profiles, resulting in greater phenotypic heterogeneity within these two groups. This pattern suggests that even isolates with nearly identical core-genome backgrounds may differ in accessory genes or resistance phenotypes, potentially due to gene gain or loss, mobile genetic elements, plasmid-associated variation, or local microevolution during environmental persistence. Thus, these subclusters should be interpreted as closely related clonal groups with limited accessory-genome diversification, rather than as completely identical bacterial populations.

Together, these findings suggest that the ecological risk of environmental *A. baumannii* in veterinary hospitals should be interpreted by integrating environmental distribution with genomic characteristics. In the present study, the potential ecological risk was not primarily reflected by widespread MDR phenotypes, but rather by the frequent recovery of isolates from high-use or high-contact environments, such as X-ray rooms, wet sites, staff uniforms, shared equipment, and other movable or frequently touched surfaces. In addition, several hospital-specific subclusters showed extremely close genetic relatedness, with ≤8 cgMLST allelic differences and 0–3 core-genome SNPs, indicating the presence of highly related isolates within individual hospitals and remaining compatible with possible local clonal persistence or limited expansion. These subclusters also carried conserved genomic determinants associated with environmental persistence, including genes related to adhesion, biofilm formation, secretion systems, oxidative-stress tolerance, and metal or biocide tolerance. The potential ecological risk of these isolates may be linked less to the broad dissemination of MDR clones and more to their ability to survive on environmental surfaces, tolerate cleaning or disinfectant-associated pressures, and redistribute across hospital spaces.

However, because paired animal clinical isolates and longitudinal epidemiological data were not available, these findings indicate potential environmental persistence risk rather than direct evidence of infection risk or confirmed transmission. Although low cgMLST distances, 0–3 core-genome SNP differences, shared hospital origin, broad within-hospital distribution, and conserved MRG profiles are compatible with possible local clonal persistence or limited expansion, this study lacked continuous temporal sampling, paired animal clinical isolates, animal referral records, personnel movement data, equipment-sharing records, and detailed contact networks. Therefore, these hospital-specific subclusters cannot be considered direct evidence of definite transmission chains or transmission direction. Future studies integrating longitudinal environmental surveillance, paired clinical and environmental isolates, detailed animal/personnel/equipment movement data, and functional validation of persistence-related determinants will be necessary to distinguish persistent environmental contamination, repeated introduction, common-source exposure, and true hospital-associated dissemination. In addition, although isolates commonly carried genes related to adhesion and biofilm formation, biofilm formation was not phenotypically assessed in this study. Future studies should combine genomic prediction with functional validation, including biofilm assays.

## 5 Conclusions

This study revealed heterogeneous environmental contamination of *A. baumannii* across veterinary hospitals in Beijing, with higher recovery rates in X-ray rooms, wet sites, staff uniforms, floors, and other shared or frequently contacted surfaces. Most isolates were not MDR, but they carried conserved resistance-, virulence-, and environmental survival-associated genomic determinants, suggesting that their ecological risk may be related more to surface survival, stress tolerance, and redistribution than to widespread MDR clone dissemination.

Population structure analyses showed both inter-hospital lineage sharing and hospital-specific near-clonal subclusters supported by cgMLST and core-genome SNP analyses. The detection of highly related isolates across multiple departments and environmental surfaces within the same hospital indicates broad within-hospital distribution of near-clonal isolates. These patterns are compatible with possible local clonal persistence or limited expansion, although this interpretation requires confirmation through longitudinal sampling, paired clinical isolates, and detailed epidemiological linkage data.

Overall, these findings highlight the need to include environmental opportunistic bacteria with strong survival potential, such as *A. baumannii*, in veterinary hospital surveillance and IPC planning, with particular attention to high-use functional areas, wet zones, staff uniforms, shared equipment, and high-touch surfaces.

## Data Availability

The datasets presented in this study can be found in online repositories. The names of the repository/repositories and accession number(s) can be found in the article/**Supplementary Material**.
